# Dental and periodontal status of 12-year-old Bulang children in China

**DOI:** 10.1186/1472-6831-14-32

**Published:** 2014-04-05

**Authors:** Shinan Zhang, Juan Liu, Edward CM Lo, Chun-Hung Chu

**Affiliations:** 1 Faculty of Dentistry, the University of Hong Kong, Hong Kong, China; 2 School of Stomatology, Kunming Medical University, Yunnan, China

**Keywords:** Caries, Children, Ethnic, Minority, China

## Abstract

**Background:**

Bulang is an ethnic minority group living in Yunnan in the southwestern part of China. There is little information pertaining to the oral health of Bulang children. This study aims to examine the dental caries and periodontal status of 12-year-old Bulang children in China and the factors affecting their oral-health status.

**Methods:**

12-year-old Bulang school children in Yunnan, China, were recruited through a multi-stage cluster sampling method. Following the recommendation of the World Health Organization, caries experiences were recorded using the DMFT index and periodontal status with the CPI index. A self-completed questionnaire was used to collect information on the background and oral health-related behaviours of the children.

**Results:**

A total of 900 children in primary schools were invited, and 873 (97%) joined the survey. Their caries prevalence was 35%. Their caries experience in mean DMFT (±SD) score was 0.6 ± 1.1, and 94% of the carious teeth had no treatment. Most children (71%) had bleeding gums, and 58% of them had calculus. Girls and those who had visited a dentist in the previous year had higher caries risk.

**Conclusions:**

Dental caries was common among the 12-year-old Bulang children in China. Most of the carious teeth were left untreated. Caries prevalence was associated with gender and dental attendance. Their periodontal condition was poor, and more than half of them had calculus.

## Background

There are persistent inequalities in dental health in many countries, although studies have suggested that tooth decay, or dental caries, has generally decreased in the past few decades, particularly in industrialized countries [1]. Ethnicity has been identified as one of the risk factors for the disparity [2]. Epidemiological studies have reported that people from specific ethnic minorities often have poor oral health status and increased risks of dental decay [3]. At present we do not have a well-accepted and comprehensive explanation regarding the ethnic disparity in oral health. A poor oral health status is often attributed to low economic status [4]. It may also be associated with unhealthy homecare practices and socio-economic barriers to dental services that are structurally maintained in ethnic groups [3,5]. Tackling oral-health inequalities effectively is therefore essential for better understanding the phenomenon and for planning and developing services orientated to reducing the problem [6].

China is a multi-ethnic country with 56 ethnic groups. Han is the predominant ethnic group. The other 55 ethnic groups account for only 8% of the total population of China. However, their combined populations was approximately 113 million in 2010, and the various populations range from a few thousand people to more than 16 million [7]. Most of these ethnic minority groups live in inland or border districts in the less-developed western regions [8]. These ethnic minorities are culturally and linguistically diverse. They speak over 80 languages, of which 30 have distinct written forms [9]. In order to secure the equality and unity of ethnic groups, the central government introduced a series of policies to promote economic and social development for them. Nevertheless, there has been little health policy formulated to provide the culturally sensitive and appropriate services that would cater to their needs, and dental health is a field that is neglected. The three national oral-health surveys, which were conducted in 1983, 1995, and 2005, documented the oral health of children geographically according to province, and ethnicity was not an essential collected category [10,11].

The history of Bulang people in China can be traced back to the 1st century [12]. Most of them (97%) live in the mountainous areas along the mid- and down-stream of the Mekong River on the southwestern frontiers of Yunnan Province, 1,500 to 2,300 meters above sea level. The social development of the Bulang people is below the national average level. It is one of the seven poor ethnic minority groups receiving special economic support from the Yunnan government [13]. The Bulang have the same ethnic origin as some ethnic groups in Myanmar, Laos, and Thailand. They speak a language that belongs to the south Asian language family, but the language has no written form. Their stable foods mainly consist of rice, maize, and beans. Their food is generally sour and hot. Drinking home-brewed wine and smoking tobacco are their pastimes. Bulang women chew betel nut and regard teeth that have been stained black with betel-nut juice to be beautiful [12]. The literature review found only one study, in 1994, reporting caries among Bulang school children [8,14]. The study used a convenience sample of 216 children and did not investigate determinants of the children’s caries status [14]. This study investigates the caries and periodontal status of Bulang school children in Yunnan China. It also examines the factors that affect the caries prevalence.

## Methods

### Selection of children and sample size

This study was approved by the Institutional Review Board of the University of Hong Kong and Hospital Authority Hong Kong West Cluster (IRB UW-11-377) and was conducted from November 2011 to May 2012. Bulang is one of the ethnic minority groups with a relatively small population (around 120,000) [12]. A sample of 12-year-old Bulang children was selected using a multi-stage, cluster sampling method in Yunnan province, China, which is where most (97%) of the Bulang people in China live [8]. Children were recruited with the help of the Bureau of Public Health and the Bureau of Education of the local government. In the first stage, 2 of the 16 districts (Xishuangbanna and Lincang) with the largest Bulang populations were selected. The 2 districts account for the majority (77%) of the total Bulang population in Yunnan [12]. Approximately 430 12-year-old Bulang children (50% of the sample size) from each of the 2 selected districts were invited. In the second stage, the counties that account for the majority (more than 50%) of Bulang people were selected from the district. Therefore, 2 of the 3 counties in Xishuangbanna (Jinghong and Menghai) and 3 of the 8 counties in Lincang (Yun, Gengma and Shuangjiang) were selected. The primary schools with Bulang children were recruited, and all 12-year-old Bulang children in the schools were invited to participate in this study. Children with parental written informed consent, of Bulang ethnic origin, and in good general health were included. Children who had major systemic diseases or syndromes or who were on long-term medications were excluded from the study. According to the third national oral-health survey conducted in 2005, the prevalence of dental caries of 12-year-old groups in western China was 25% [10]. This study estimated the caries prevalence to be 25%. Standard error was set to 1.5% and the response rate was assumed to be 90%. The number of the 12-year-old school children invited needed to be at least 856.

### Questionnaire survey

Before the main survey, the examiner (SZ) visited each primary school and discussed the protocol with the teacher in charge of this study. A self-completed questionnaire was delivered to the children in schools under the supervision of a research assistant. A written set of instructions was given to the students on answering the questionnaire that was later collected with the help of teachers. The questionnaire was used to collect information on the backgrounds (gender, address), oral health-related behaviours (snacking habit, tooth-brushing practice, and dental visits), and oral-health knowledge of the surveyed students, which was used in previous studies [15-17]. Oral-health knowledge was measured with standardized questions pertaining to the causes and prevention of dental diseases and gum diseases, and a dental-knowledge score was constructed.

### Clinical examination

All the participating children underwent clinical examinations, which were conducted by a trained dentist in the primary school using a 0.5 mm ball-ended CPI probe and a disposable dental mirror attached to an intra-oral LED light. Dental caries was diagnosed and recorded based on the methods and criteria recommended by the World Health Organization [18]. Caries was detected visually if a lesion had an unmistakable cavity, undermined enamel, or a detectably softened floor or wall. Signs of early caries, such as white- or brown-spot lesions, rough surfaces, or fissures sticky to probing but without a detectably softened floor or wall were not diagnosed as dental caries.

‘Caries experience’ is an indication of history of exposure to tooth decay. The DMFT index was used to record the caries experience of the permanent dentition: ‘D’ stands for decayed tooth, ‘M’ denotes missing tooth due to decay, and ‘F’ represents filled tooth [18]. The ‘PA’ index, which is modified from the ‘PUFA’ index, was used to record the clinical consequence of caries disease [19]. The ‘P’ denotes an untreated carious tooth with visible pulp involvement. This was recorded when the pulp chamber was visible or only the roots or root fragments were left. The ‘A’ denotes an untreated carious tooth with a visible apical infection that can be in the form of an abscess or fistula. This was recorded when pus containing swelling (i.e., an abscess) or being released in the sinus tract (i.e., a fistula) related to a tooth with pulp involvement was present. The periodontal status was assessed using the community periodontal index (CPI) on the indexed teeth. The score could be 0 (healthy–absence of gingival bleeding), 1 (gingival bleeding), and 2 (presence of supra or sub-gingival calculus) [18]. The index teeth were the four first permanent molars (16, 26, 36, and 46), upper-right central incisor (11) and lower-left central incisor (31). Six sites (mesio-buccal, mid-buccal, disto-buccal, mesio-lingual, mid-lingual and disto-lingual) were examined in each tooth, and the worst condition was recorded. Duplication examinations were performed on 10% of the participants to assess the intra-examiner agreement of assessing DMFT, PA, and CPI using Cohen’s Kappa value.

### Data entry and analysis

Data processing and analysis were performed by the Statistical Package for Social Sciences, version SPSS 20.0. The differences in the means (mean DMFT scores, mean ‘PA’ scores, and the mean proportion of the periodontal condition) were analysed by an independent t-test (two groups) or ANOVA (more than two groups). The Chi square test was used to compare proportions. Binary logistic regression was performed to assess the effects of the independent variables studied, including the demographic background of the child, the child’s oral health-related behaviours, and knowledge of the child’s dental caries prevalence. The dependent variable was the presence of dental caries or lack thereof, and a backward stepwise procedure was used to remove the variables that were not statistically significant. The statistical significance level for all tests was set at 5%.

## Results

A total of 900 children were invited from 58 primary schools, and 873 children joined the survey. The response rate was 97%. There were 439 (50%) boys and 434 (50%) girls. The kappa values for scoring ‘DMFT’, ‘PA’, and CPI were 0.98, 0.97, and 0.90, respectively.

The caries experience, caries prevalence, clinical consequences of caries disease, and its prevalence amongst the Bulang children are shown in Table 1. There were 35% of the subjects (N = 301) who had caries experience. The mean DMFT (±SD) score was 0.6 ± 1.1. The majority of decayed teeth (94%) were left untreated, and the highest DT score was found in a child with 8. Higher DMFT scores were found in the girls than in the boys (0.8 ± 1.2 vs. 0.5 ± 0.9; p = 0.004). Girls also had higher DT and FT scores than those of the boys. Approximately one third (32%) of the carious teeth had progressed to pulp infection (PA > 0). The mean PA (±SD) score for those children who had caries was 0.4 ± 0.7. A higher proportion of pulpally involved teeth (PA > 0) was found among the girls than the boys (13% vs. 8%, p = 0.010).

**Table 1:** **Dental caries status according to gender (N** = **873)**

**Gender**	**N (%)**	**% caries**	**DMFT (SD)**	**DT (SD)**	**% PA**	**% A**	**PA (SD)**	**A (SD)**
All	873 (100%)	35%	0.6 (1.1)	0.6 (1.1)	11%	1.1%	0.2 (0.5)	<0.1 (0.1)
Boys	439 (50%)	31%	0.5 (0.9)	0.5 (1.0)	8%	0.9%	0.1 (0.4)	<0.1 (0.1)
Girls	434 (50%)	39%	0.8 (1.2)	0.7 (1.2)	13%	1.4%	0.2 (0.5)	<0.1 (0.1)
p-value		0.013	0.004	0.001	0.010	0.513	0.024	0.513

Table 2 shows the tooth-brushing frequencies and snacking and dental-visit behaviours of the children according to gender. Most of the children (90%, N = 768) brushed their teeth at least once daily, and there were more girls (30%, N = 126) who brushed at least twice daily than there were boys (20%, N = 83). A daily snacking habit was common, and no significant difference was found between boys and girls. There was no significant difference in the oral-health related knowledge between boys and girls. Table 3 shows the caries prevalence and variables studied. Results of logistic regression show that the prevalence of dental caries were significantly related to the dental visit habit (OR: 1.801, 95% C.I: 1.182-2.744). The odds for girls having caries experiences (DMFT > 0) were 1.447 times more than for boys (Table 4).

**Table 2:** **Toothbrushing habit**, **snacking habit and dental visit behavior according to gender**

**Factor (No.)**	**All (%)**	**Boys (%)**	**Girls (%)**	**p-value**
Brushing twice or more daily (N = 843)				0.001
Yes	209 (25%)	83 (20%)	126 (30%)	
No	634 (75%)	335 (80%)	299 (70%)	
Eating snacks daily (N = 837)				0.557
Yes	459 (55%)	225 (54%)	234 (56%)	
No	378 (45%)	193 (46%)	185 (44%)	
Visiting dentist within last year (N = 852)				0.124
Yes	105 (12%)	45 (11%)	60 (14%)	
No	747 (88%)	380 (89%)	367 (86%)	

**Table 3:** Caries prevalence and variables studied

**Independent variable**	**Group (N)**	**DMFT >** **0 (n, %)**	**p**-**value**
Gender	Boys (439)	134, 31%	0.013
	Girls (434)	167, 39%	
Location	Towns (424)	154, 36%	0.266
	Villages (449)	147, 33%	
Brushing twice or more daily	Yes (209)	74, 35%	0.674
	No (634)	213, 34%	
Eating snacks daily	Yes (459)	160, 35%	0.763
	No (378)	128, 34%	
Visiting dentist within last year	Yes (105)	50, 48%	0.002
	No (747)	241, 32%	

**Table 4:** Relationship between caries prevalence and selected independent variables

**Independent variables**	**DMFT > 0 (n, %)**	**Odds ratio**	**95% C.I**	**p-value**
Gender				
Girls	164,38%	1.447	1.104-1.976	0.006
Boys^a^	128,30%			
Visited a dentist within last year				
Yes	50, 48%	1.801	1.182-2.744	0.009
No^a^	241,32%			
(Constant)		0.399		<0.001

^a^Reference group.

29% of the children had healthy gums (highest CPI = 0) (Table 5). Most children (71%) had bleeding gums, and more than half of them (58%, N = 505) had calculus (Highest CPI = 2). The mean number of sextants (SD) with calculus was 1.1 (1.2). Boys had a higher prevalence of calculus than the girls (64% vs. 52%, p < 0.001).

**Table 5:** **Periodontal status according to gender** (**N** = **873**)

	**All**	**Boys**	**Girls**	**P value**
No. (%) of children				
Highest CPI = 0	257 (29%)	111 (25%)	146 (34%)	0.007
Highest CPI = 1	111 (13%)	48 (11%)	63 (15%)	0.112
Highest CPI = 2	505 (58%)	280 (64%)	225 (52%)	<0.001
Mean (SD) number of sextants:				
Healthy (CPI = 0)	4.6 (1.3)	4.4 (1.3)	4.7 (1.2)	<0.001
Bleeding only (CPI = 1)	0.3 (0.6)	0.4 (0.7)	0.3 (0.6)	0.318
With calculus (CPI = 2)	1.1 (1.2)	1.2 (1.2)	1.0 (1.1)	0.001

## Discussion

A recent review concluded that dental caries in children is one of the major health problems and has become a huge burden in China [20]. Disease levels are increasing rapidly in developing countries, particularly as a result of a growing consumption of sugars and inadequate exposure to fluorides. To ensure effective delivery of interventions and optimal allocation of resources, an epidemiological survey based on ethnic groups is crucial to identify the high-risk groups in a multicultural society.

This study required a relatively large sample size (N = 856) of 12-year-old Bulang children, which was more than 40% of all 12-year-old Bulang children (estimated to be 2,000) in Yunnan. The Bulang people are widely distributed in Yunnan province, but the majority of them are distributed in the rural areas of certain districts. Therefore, a pragmatic approach for recruiting the participant is by using a purposeful sampling method. This sampling method is convenient and cost-effective, but the sample is not representative of the entire population. One of the major reasons for the high response rate is the support from the local education and public-health bureau. The pre-survey visits to the schools were also important. Apart from developing effective communication, invitation letters and consent forms were delivered to parents through the schools. This provided sufficient time for the schools and parents to understand the aim and method of the study. A simple and easily understood questionnaire was used to guarantee the quality of the study, which includes a child’s oral-health–related behaviours and background information. Thus, a relatively complete understanding of participants’ oral behaviours health and social contexts could be expected. However, some questions such as about snacking on sweets daily could be too general for inference. This study kept this question because it had been used in previous studies, and therefore to keep the question facilitates comparisons. Nevertheless, more depth is definitely necessary if the study aims to elaborate on these factors as possible underlying reasons for the serious caries situation.

The prevalence of dental caries and the mean DMFT score of the Bulang children in this study was higher than that of the 12-year-old children reported in the third national oral-health survey [10]. This may be because most Bulang people live in the subtropical zone, where sugar is produced. Convenient access to sugar and the hot climate are likely to promote its consumption in the form of sweets and soft drinks. Meanwhile, as the interflow with Han people increases, more refined foods are becoming more readily available. On the other hand, use of dental services in rural areas is very limited, and this exacerbates the children’s dental problems. Therefore, strategies to promote oral health and prevent dental caries must be introduced.

Although dental caries is caused by plaque on the tooth surface, strong clinical evidence that toothbrushing per se is effective in caries prevention is lacking [21]. Results of this study agree with such a conclusion. It was observed that girls brush their teeth more frequently than boys do, but girls had higher caries rate than had the boys. This finding also concurs with that of the national surveys [10]; it may be because permanent teeth generally erupt earlier in girls than in boys.

These results show that a majority of the children had not visited dentists within the last year. However, a higher caries rate was found in the children who had visited dentists. This may be due to the common problem-oriented dental-care-seeking behaviour in China. It is plausible that children were brought to visit dentists for pain and infection due to dental caries. The limited availability of dental-care resources may be another reason. Dental clinics are extremely uncommon in villages, and therefore patients must go to towns or their county dentists to treat dental diseases. The dentist to population ratio is around 1:30,000 in China and the number of dentists is even fewer in rural areas [22]. It is crucial for the government to train and encourage more dentists to work in rural areas. Effort should be made to promote primary dental care. Community oral-health programs can be implemented to help ensure that those needs regarding dental caries are met [23].

The periodontal health status of the 12-year-old children in this survey was unsatisfactory, and most of them had gingivitis, which was worse than that of the third national oral-health survey. However, a somewhat high number of children claimed to brush their teeth at least once a day. This inconsistency between found periodontal health and reported dental hygiene practice could be a result of the children over-reporting their tooth brushing habits or having not acquired the proper brushing techniques. Gingival health can be restored through proper oral hygiene practice. Motivation to implement the instructions given for oral healthcare and regular reinforcement are essential in promoting children’s oral health [24]. School-based oral health programs, which were proved feasible and effective in promoting the dental and gingival health in China, can be carried out in these areas [25].

## Conclusions

Dental caries was common among the 12-year-old Bulang children in China. Many of the decayed teeth were left untreated. The caries prevalence was associated with gender and dental attendance. The prevalence of gingival inflammation and calculus was high.

## Competing interests

The authors declare that they have no competing interests.

## Authors’ contributions

SZ performed the survey and ECML, JL and CHC supervised this work. The 4 authors contributed equally to preparation of the manuscript. All authors read and approved the final manuscript.

## Authors’ information

Dr. Shinan Zhang is a PhD student in the Faculty of Dentistry. Professor Edward C. M. Lo, Dr. Juan Liu and Chun-Hung Chu are supervisors of Dr. Zhang.

## Pre-publication history

The pre-publication history for this paper can be accessed here:

http://www.biomedcentral.com/1472-6831/14/32/prepub
